# Mercury transport and human exposure from global marine fisheries

**DOI:** 10.1038/s41598-018-24938-3

**Published:** 2018-04-30

**Authors:** Raphael A. Lavoie, Ariane Bouffard, Roxane Maranger, Marc Amyot

**Affiliations:** Groupe de Recherche Interuniversitaire en Limnologie et environnement aquatique (GRIL), Département de Sciences Biologiques, Université de Montréal, Pavillon Marie-Victorin, CP6128, Succ. Centre-ville, Montréal, Québec, H3C 3J7 Canada

## Abstract

Human activities have increased the global circulation of mercury, a potent neurotoxin. Mercury can be converted into methylmercury, which biomagnifies along aquatic food chains and leads to high exposure in fish-eating populations. Here we quantify temporal trends in the ocean-to-land transport of total mercury and methylmercury from fisheries and we estimate potential human mercury intake through fish consumption in 175 countries. Mercury export from the ocean increased over time as a function of fishing pressure, especially on upper-trophic-level organisms. In 2014, over 13 metric tonnes of mercury were exported from the ocean. Asian countries were important contributors of mercury export in the last decades and the western Pacific Ocean was identified as the main source. Estimates of *per capita* mercury exposure through fish consumption showed that populations in 38% of the 175 countries assessed, mainly insular and developing nations, were exposed to doses of methylmercury above governmental thresholds. Our study shows temporal trends and spatial patterns of Hg transport by fisheries. Given the high mercury intake through seafood consumption observed in several understudied yet vulnerable coastal communities, we recommend a comprehensive assessment of the health exposure risk of those populations.

## Introduction

Anthropogenic emissions of mercury (Hg), largely a function of small-scale gold mining, coal burning, incineration, and other mining activities, have increased atmospheric and surface ocean concentrations three to five and two to three-fold respectively since the preindustrial era^1–3^. Hg inputs to the ocean is primarily through wet and dry atmospheric deposition of divalent inorganic Hg (Hg^II^), which can be reduced to the volatile gaseous elementary Hg (Hg^0^) or methylated to form the highly bioaccumulative methylmercury (CH_3_Hg; hereafter MeHg)^2,4^. MeHg is of particular interest, since it is a potent neurotoxicant^5^ that biomagnifies along aquatic food chains^6^ often reaching hazardous concentrations in fish-eating wildlife^7^ and human populations^8^. In addition to abiotic fluxes, transport of contaminants through animal movement, known as biotransport, can also alter local and regional contaminant fluxes^9^. Human-mediated transport through fisheries has altered the flow of elements^10^ and contaminants^11^. The contribution of Hg removal through fisheries has been included in previous global and regional Hg cycle models and has been found to be relatively modest^1,12,13^. However, a detailed spatial, temporal and taxonomical analysis of Hg export by global fisheries has yet to be done. Intensity of fishing pressure has increased since the 1950s as a result of expansion into the high seas and deeper waters facilitated by technological innovations^14–16^. With over 167 million tonnes (Mt) of fish harvested annually through capture and aquaculture from marine and inland waters^17^, biomagnifying contaminants such as Hg can be transported by fisheries and we hypothesize that there are regional patterns and temporal trends related to this transport. Seafood consumption can be high in certain countries and can therefore lead to a higher exposure to MeHg, the toxic form of Hg. Species guilds can be particularly important for defining exposure^18^ as higher trophic level species contain more Hg^6^. We hypothesize that exposure will greatly vary among countries based on intake rate and fish species consumed. The objectives of this study were to (1) quantify the amount of THg and MeHg transported from the ocean through marine fisheries between 1950 and 2014 and (2) assess human exposure to MeHg through seafood consumption in 175 countries between 1961 and 2011.

## Temporal trends of Hg export from fisheries

**Figure 1:**
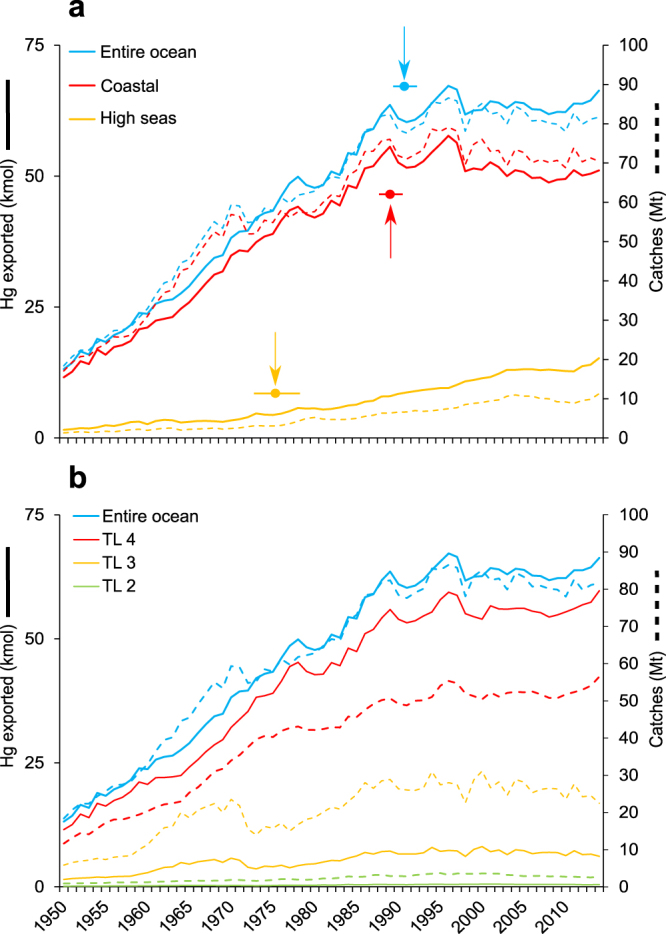
Temporal trends (1950‒2014) of marine fisheries catches (dashed lines) and mercury exported (full lines). (**a**) Entire ocean (blue) and coastal (red) and high seas (orange) with breakpoint (circle) ± standard error (horizontal line). See Supplementary Table 1 for results of the segmented regressions. (**b**) Entire ocean (blue) and trophic levels (TL) rounded to the nearest integer: 4 (red), 3 (orange), and 2 (green).

Hg exported from oceans due to marine fisheries harvest (invertebrates, fish, reptiles, and marine mammals) was calculated by pairing catch time series data (1950–2014) from the Food and Agriculture Organization of the United Nations (FAO) with Hg concentrations from the literature (see methods). MeHg concentrations were estimated based on trophic level and taxa. Total Hg (THg) exported from the entire ocean increased steadily from 13.2 kmol in 1950 until a breakpoint in 1991 (± 0.7 yr SE; see Supplementary Table 1 for segmented regressions) followed by a plateau reaching 66.3 kmol in 2014 (Fig. 1a). MeHg export followed a similar temporal pattern as THg and varied from 6.3 kmol in 1950 to 37.8 kmol in 2014 (Supplementary Fig. 1). We compared the 2014 THg export from fisheries with a global estimate of geochemical Hg fluxes of loss processes from the ocean (to the atmosphere: 10,000–14,700 kmol a^−1^ of Hg° and to the sediments: 1,100 kmol a^−1^ of THg)^1^. Fisheries export represented only 0.4–0.6% of the total loss. Most of the THg removed through fisheries was from coastal zones throughout the entire studied period. However, since 1989 (±0.6 yr SE) the amount exported from the coasts has been on the decline (Fig. 1a; Supplementary Table 1). Unsustainable fishing practices have resulted in a more precipitous decline in predatory fish biomass in coastal ecosystems when compared to the open ocean^19^. To compensate for relative loss in catch from the coasts, the proportion exported from high seas has increased since 1975 (±1.3 SE yr; Fig. 1a; Supplementary Table 1). In order to meet the demands of the industry, fisheries have not only intensified through expansion into the high seas^15^ but have also extended into deeper waters^14^.

The vast majority of THg exported from the entire ocean was from upper-trophic-level species (Fig. 1b; Supplementary Fig. 2). This result was not only driven by a higher biomass of catches, but also by higher Hg concentrations at the top of the food chain due to MeHg biomagnification^6^. Species from the group “Tunas, bonitos, and billfishes” are high in the food chain (trophic level (TL) = 4.3 ± 0.7 SE; *n* = 47^20^; Supplementary Table 2) and consequently had a high average whole-body THg concentrations in our study (0.38 ± 0.39 standard deviation weighted for sample size (SD_w_) *μ*g g^−1^; Supplementary Table 3) while species from the group “Oysters” have a low TL (2.1 ± 0.13 SE^20^) and therefore a low THg concentration (0.015 ± 0.014 SD_w_ *μ*g g^−1^). Between 1950 and 2014, species of TL 4 contributed to 63 ± 4% SD of total catches in biomass, but contributed to 88 ± 2% SD of the THg exported due to their high THg concentrations. On the other hand, species of TL 2 contributed to 33 ± 4% SD of total catches throughout the time series, but their low THg concentrations resulted is a contribution of only 12 ± 2% SD of the total THg exported. Thus, mercury export from the ocean is not only a function of total catch like in other elemental cycles^10^ but also of species composition. Upper-trophic-level species, with the most significant Hg burden, have a high consumer value and catches have been constant or are even on the rise in the last decade (Fig. 1b) due to expansion of this prized fishery into the high seas.

Overall, the Hg export values presented here are likely underestimations since catches are approximately 50% higher than what is reported by the FAO due to the lack of information or poor accountability of harvest from small-scale, artisanal, and illegal fisheries^21^.

## Spatial patterns of Hg export from fisheries

**Figure 2:**
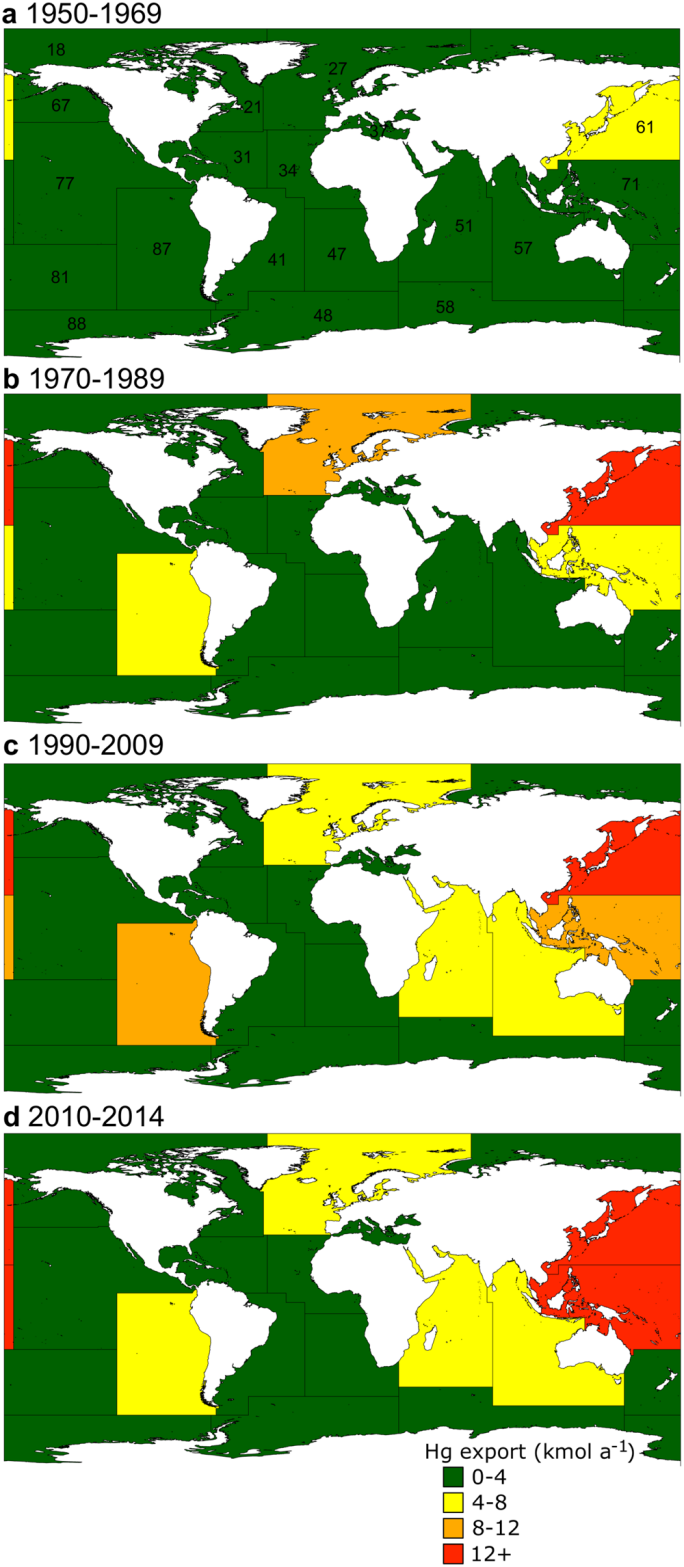
Mercury export from the ocean. Mean total Hg export in (**a**) 1950–1969, (**b**) 1970–1989, (**c**) 1990–2009, and (**d**) 2010–2014 resulting from catches of fishes, molluscs, crustaceans, reptiles, and mammals for each MFA (numbers are provided in panel (a) and corresponding names are found in Supplementary Table 7). Standard deviations of means for each period are found in Supplementary Fig. 4. Maps were created in ArcGIS version 10.3.1 (http://desktop.arcgis.com/).

THg removed from the ocean via fisheries has not only changed over time as a function of increased capture and changes in species composition, but also over space. Among the FAO delimited Major Fishing Areas (MFA), the Northwest Pacific (MFA #61) was the greatest THg exporter throughout the time series, while the Northeast Atlantic (MFA #27), the Southeast Pacific (MFA #87), and the Western Central Pacific (MFA #71) successively took the second rank between 1950–1986, 1987–1997, and 1998–2014, respectively (Fig. 2; Supplementary Fig. 3). In general, THg export from a given MFA was closely related to annual capture, but some regions did not reflect this trend due to the species composition of catches. For example, THg export in 2014 was similar for the Northwest Pacific and Western Central Pacific, although catches differed by almost a factor of two, at 22.0 and 12.8 Mt, respectively. In the Western Central Pacific region, “Tunas, bonitos, billfishes” was the dominant group of species and accounted for 27% of catches, but 43% of THg export in 2014 due to their high TL. In contrast, the low trophic level group “Herrings, sardines, anchovies” ranked 4^th^ in terms of catches in the Northwest Pacific in 2014 and accounted for 10% of total catches, but only 3% of THg export. Hence, composition of catches should be considered in addition to the total amount when considering THg export.

**Figure 3:**
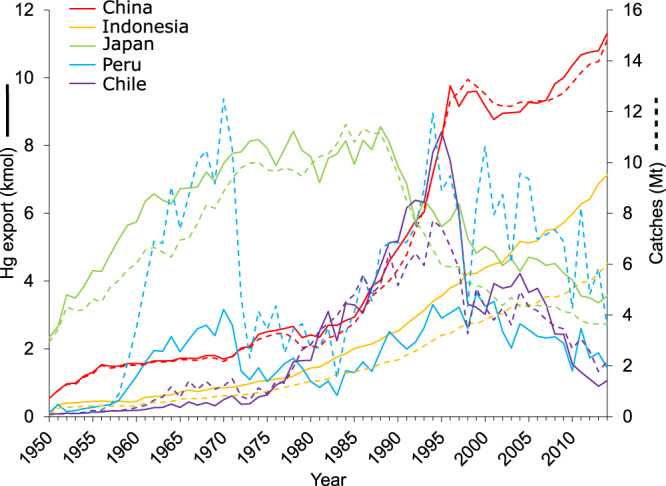
Temporal trends (1950‒2014) of catches (dashed lines) and Hg exported (full lines). China (red), Indonesia (orange), Japan (green), Peru (blue), and Chile (purple).

In 2014, the overall THg export was highest in Northwest Pacific (16.5 kmol), Western Central Pacific (15.3 kmol), and Eastern Indian Ocean (MFA #57; 7.8 kmol; Fig. 2; Supplementary Fig. 3), which combined, accounted for 60% of global total. These zones are where coastal population density is high and where marine resources represent a significant proportion of the human diet, harvest or trade commodities^8,22,23^. Japan was historically the largest exporter of THg through fisheries, but a decline after 1990 coincided with a rapid rise by China, Chile (followed by a sharp decline after 1995), and Indonesia (Fig. 3). In 2014, China (11.3 kmol), Indonesia (7.1 kmol), and Japan (3.6 kmol) contributed to 33% of the global THg exported over the entire ocean (Fig. 3). Although a significant fraction of capture by these countries serves to feed their own populations, these nations play an active role in trading catches around the world (with China being by far the main exporting country in 2014)^17^, thus influencing the Hg flow among countries worldwide and the relative MeHg exposure to humans.

## Human Hg exposure from seafood consumption

For many countries, marine fishes are the main protein source and therefore high consumption rates could result in a significant risk of intake of MeHg, the toxic and bioaccumulative form of Hg^17,18^. In order to estimate exposure risk as a function of fish availability through trade flows, we calculated the mean *per capita* MeHg weekly intake (WI) for each country and compared these estimates to the provisional tolerable weekly intake (PTWI) of 1.6 *μ*g kg^−1^ of body mass (BM) week^−1^. The latter PTWI is considered a safe consumption threshold to protect developing fetuses^24^ and is therefore technically used as a baseline for overall safe consumption for human health. Potential WI was based on available fish supply for consumption (i.e., edible fraction of fisheries harvests) *per capita* in each country after considering imports and exports through trade and after removing non-food uses and waste^22^.

Interestingly, an overall increase in global WI of Hg was observed over time, with estimated potential indices in some countries being remarkably high (Table 1). Indeed, for the period between 2001 and 2011, we estimated that 38% of the countries assessed here (66/175) potentially exceeded the recommended PTWI of MeHg of 1.6 *μ*g kg^−1^ week^−1^ with 42% of those (28/66) being Small Island Developing States (SIDS) and/or Least Developed Countries (LDC; Table 1; Supplementary Table 4 for an extended list). Potential WI reached a value of 23.1 ± 5.3 *μ*g kg^−1^ week^−1^ for the Maldives, 14 times higher than the recommended PTWI (Table 1). A recent global meta-analysis conducted on women and infants from seafood-consuming populations also found high risks to populations of several countries^8^. The authors estimated that 34% of populations (239 subpopulations from 43 countries) exceeded the PTWI^8^. This level of risk was particularly located in tropical riverine populations near gold mining sites (85% of populations > PTWI) and traditional Arctic populations (75% of populations > PTWI)^8^. Furthermore, coastal populations from Southeastern Asia, the Western Pacific, and the Mediterranean were reported to have an elevated risk^8^ which is in accordance with the MFAs we found to export the greatest amount of MeHg through fishing.

**Table 1:** *Per capita* potential MeHg (mean ± standard deviation; SD) weekly intake (WI; *μ*g of MeHg kg of body mass^−1^ week^−1^) of marine fish and seafood of the top countries and the world average for each decade between 1961 and 2011 based on food supply^22^.

Country	Status	1961–1970	1971–1980	1981–1990	1991–2000	2001–2011
Maldives	SIDS	**9**.**5** ± 6.6	**14**.**1** ± 2.5	**20**.**1** ± 4.4	**24**.**0** ± 4.3	**23**.**1** ± 5.3
Kiribati	SIDS, LDC	**4**.**7** ± 0.4	**5**.**0** ± 0.2	**6**.**4** ± 1.5	**7**.**5** ± 0.3	**8**.**0** ± 0.8
Iceland		**4**.**7** ± 1.6	**7**.**6** ± 2.3	**7**.**8** ± 2.0	**6**.**2** ± 1.7	**7**.**5** ± 1.6
Malaysia		**2**.**6** ± 0.3	**3**.**9** ± 1.0	**5**.**0** ± 0.3	**6**.**0** ± 0.7	**6**.**4** ± 0.4
Samoa	SIDS, LDC	**4**.**1** ± 0.3	**3**.**5** ± 0.8	**4**.**4** ± 0.5	**5**.**2** ± 1.6	**6**.**4** ± 0.8
French Polynesia	SIDS	**5**.**2** ± 0.7	**3**.**9** ± 0.6	**3**.**8** ± 0.4	**5**.**0** ± 0.4	**5**.**0** ± 0.1
Lithuania		NA	NA	NA	**2**.**5** ± 0.9	**4**.**8** ± 0.4
Japan		**5**.**3** ± 0.2	**5**.**9** ± 0.2	**6**.**0** ± 0.2	**5**.**4** ± 0.4	**4**.**8** ± 0.5
Barbados	SIDS	**2**.**9** ± 0.5	**3**.**3** ± 0.6	**4**.**0** ± 0.7	**4**.**0** ± 0.4	**4**.**8** ± 0.2
Republic of Korea		1.4 ± 0.3	**2**.**5** ± 0.5	**3**.**0** ± 0.4	**3**.**6** ± 0.8	**4**.**7** ± 0.1
Grenada	SIDS	**3**.**2** ± 0.6	**4**.**4** ± 0.5	**3**.**6** ± 0.8	**3**.**1** ± 0.7	**4**.**6** ± 0.4
Vanuatu	SIDS, LDC	**4**.**6** ± 0.4	**5**.**6** ± 1.0	**3**.**6** ± 0.4	**3**.**5** ± 0.4	**4**.**3** ± 0.3
Fiji	SIDS	**2**.**2** ± 0.3	**2**.**9** ± 0.6	**3**.**3** ± 0.8	**2**.**2** ± 0.5	**4**.**1** ± 0.7
Sao Tome and Principe	SIDS, LDC	**1**.**7** ± 0.7	1.3 ± 0.6	**3**.**9** ± 1.0	**4**.**1** ± 0.6	**3**.**9** ± 0.2
Philippines		**3**.**4** ± 0.5	**4**.**2** ± 0.4	**3**.**6** ± 0.2	**3**.**7** ± 0.3	**3**.**9** ± 0.2
China (Hong Kong)		**2**.**4** ± 0.5	**2**.**9** ± 0.4	**2**.**3** ± 0.3	**3**.**3** ± 0.3	**3**.**9** ± 0.2
World average		1.3 ± 0.1	1.5 ± 0.0	**1**.**6** ± 0.0	**1**.**6** ± 0.0	**1**.**7** ± 0.0

Countries were ranked according to decreasing WI values of the 2001–2011 period. Economic status from the United Nations^58^ are SIDS: Small Island Developing States, LDC: Least Developed Countries. NA, not applicable (values only available since 1988^22^). Values exceeding the recommended 1.6 *μ*g kg^−1^ week^−1^ provisional tolerable weekly intake (PTWI^24^) are shown in bold. See Supplementary Table 4 for WI values for all years and countries.

Our PTWI estimates are based on available rather than consumed fish, and could therefore be considered an overestimation. However we argue that this is most likely not the case. First, the values reported here are national means, and some indigenous coastal populations consume on average 15 times more fish *per capita* than non-indigenous country populations^23^. In addition, the MeHg intake in our study excludes consumption from freshwater fish. The global marine fisheries corresponds to 49% of the total aquatic food production (capture and aquaculture from marine and inland)^17^, which suggests that the MeHg PTWI reported here could be even higher in some cases.

On the other hand, MeHg bioaccessibility and bioavailability have been traditionally assumed to be 95–100%, but recent evidence suggests that cooking methods and co-ingestion of other food items (e.g., tea) can reduce bioaccessibility^25–28^. This casts doubt on the validity of the widely used PTWI of 1.6 *μ*g kg^−1^ week^−1^. Moreover, exposure and risk may differ widely among countries depending on cultural habits, fish species consumed, ethnicity, and genetics^25–30^. A recent study suggested that country-specific seafood consumption advisories should be used for risk assessment and to protect the population^30^. Finally, benefits (e.g., intake of omega-3 polyunsaturated fatty acids) of fish intake generally exceed the potential risks due to Hg toxicity in adults. However, caution should be taken on amounts and species consumed, especially for pregnant women and nursing mothers^31^. Additional studies are needed to further assess *in situ* Hg consumption and exposure in countries and their subpopulations most at risk.

Our study shows spatial patterns and temporal trends of human-mediated transport of Hg through marine fisheries. The amount of Hg exported via fisheries is modest compared to other global ocean loss estimates. However, given the influence of global marine fisheries on MeHg exposure to humans, the abundance of seafood products available for consumption worldwide and the MeHg concentration they contain suggests that many populations, primarily from understudied developing and insular countries, could be at risk of exposure above the safety threshold. Empirical studies based on consumed (rather than available) seafood and exposure in susceptible countries outlined in this study are needed to ensure human wellbeing with regards to protection against MeHg toxicity and to balance the risks and benefits associated with seafood consumption. Our study is especially relevant in the context of the global treaty of the Minamata Convention on Mercury, whereby objective is to protect human health and the environment from adverse effects of anthropogenic Hg^32^.

## Methods

In summary, we estimated the global amount of Hg exported from oceans via marine fisheries and the human exposure to Hg from seafood consumption worldwide. We used a database of Hg in marine seafood, which was then paired to biomass: (1) marine catches in the case of Hg export and (2) marine fish available for food consumption *per capita* in 175 countries in the case of human exposure.

### Hg export via fisheries

The annual catch of marine fisheries from 1950 to 2014 was determined using the software FishStatJ from the FAO^33^. Catches were calculated by MFA and categorized into 41 groups based on the International Standard Statistical Classification of Aquatic Animals and Plants (ISSCAAP^17,33^; Supplementary Table 3). Inland waters catches as well as marine plant, coral, sponge, and pearl catches were excluded from the analysis. Catch values are reported in metric tonnes (1,000 kg) in the FAO database, except for 3 ISSCAAP groups: “Blue-whales, fin-whales” (baleen whales or mysticetes), “Sperm-whales, pilot-whales” (toothed whales or odontocetes) and “Eared seals, hair seals, walruses” (pinnipeds), which are reported in annual capture of individuals rather than by mass. A mean mass equivalence of 43, 1.29 and 0.231 tonnes were respectively used to transform values from number of individuals into tonnes for these groups (see Supplementary Table 5 for a literature review of mass values and references).

Mercury concentrations were assigned to each of the 41 ISSCAAP groups. Concentrations (mainly from muscle tissues) were derived primarily from a modified version of the online Seafood Hg Database from Karimi and colleagues^34^ available online: http://www.stonybrook.edu/commcms/gelfond/fish/database.html. Hg concentrations from market fish were excluded from the Seafood Hg Database because their origin was unknown. Hg concentrations of farmed fish from aquaculture were also excluded from the Seafood Hg Database. Moreover, farmed fish were not taken into consideration in terms of biomass in the calculation of Hg exported. The reason is that they represent a net null Hg budget when comparing Hg exported from vs. imported into the system (i.e., Hg accumulated during their growth period and exported from the system via capture is equal to Hg introduced to the system by feeding those fish during that period). Including farmed fish from the aquaculture sector could also lead to a double counting of Hg transport since fish feed often contain seafood products taken from the ocean (e.g., fish meal or oil)^35,36^. In addition to the Seafood Hg Database^34^, we searched the scientific literature to find Hg values for missing ISSCAAP groups, including some marine mammals, turtles, and invertebrates. An additional 2,952 data points from 68 taxa in 40 studies was added to the Seafood Hg Database^34^, which resulted in a total sample size of 52,224 from 416 taxa taken from 260 studies. This data was used to pair Hg concentrations to annual catches for each ISSCAAP group (see Supplementary Table 3 for a literature review of Hg concentrations and references). Mean THg concentrations in fish muscle for each ISSCAAP group were transformed into whole body concentrations according to the equation in Peterson and colleagues^37^:

1 $${{\rm{l}}{\rm{o}}{\rm{g}}}_{10}[whole\,fish\,Hg]=({{\rm{l}}{\rm{o}}{\rm{g}}}_{10}[muscle\,Hg]-\,0.2545)/1.0623$$

For whales, tissue Hg concentrations were converted to whole body concentrations using several tissues of odontocetes^38^ and mysticetes^39^ multiplied by the proportion of each tissue mass to the whole body mass^40^:

2 $$Whole\,whale\,Hg={\rm{\Sigma }}({C}_{i}\times {M}_{i})$$

where *C*_*i*_ is the mean Hg concentration (*μ*g g^−1^ w.w.) and *M*_*i*_ is the mean mass (in tonne) of the *i*^th^ tissue. For odontocetes, muscle, bone, and viscera concentrations were used to calculate whole body concentrations. For mysticetes, muscle, kidney, liver, spleen, and lung concentrations were used. For pinnipeds, we used the estimate of Yamamoto and colleagues^41^ who measured Hg in 15 tissues and calculated a whole body burden. For invertebrates, reported concentrations were assumed to be equal to whole body concentrations.

Weighted means (Hg_w_), standard deviations (SD_w_; as calculated in Karimi and colleagues)^34^, and 95% confidence intervals weighted for sample size (CI_w_) were calculated for each ISSCAAP group to estimate error for the total Hg exported from the ocean (Supplementary Fig. 1). Mean THg concentrations in whole organisms from each ISSCAAP group were multiplied by the annual catch of each group to estimate the THg exported by marine fishing. Coastal THg removal by marine fisheries was estimated by removing oceanic taxa (58 epipelagic species and 62 deep-water species)^42^ as their captures were made outside the continental shelf area (considered to be between 0 and 200 m in depth)^42,43^.

To estimate the MeHg export by fisheries, we used percent THg as MeHg values for whole organisms rather than for edible portions. Consequently, %MeHg values are lower than the generally accepted 95% for edible portions. We used a %MeHg of 58% for whole fish (*n* = 39)^44^, 46% for whole invertebrates (*n* = 462)^44–46^, and 39% for various tissues of turtles (*n* = 9)^47^. For marine mammals, we estimated the whole body burden of MeHg based on equation (2) using published %MeHg^48,49^ resulting in an average of 19% of THg as MeHg (*n* = 206). Trophic levels for each ISSCAAP group were obtained from FishBase, an online aggregate database^20^. The annual Hg export by marine fishing was calculated for each MFA and for the entire ocean from 1950 to 2014. Averages for each MFA for periods 1950–1969, 1970–1989, 1990–2009, and 2010–2014 are shown in Fig. 2 and standard deviations are shown in Supplementary Fig. 4. A recent meta-analysis by Bonito and colleagues^50^ on marine fish found no spatial differences in Hg concentrations among oceans (East Pacific Ocean, West Pacific Ocean, Atlantic Ocean, Indian Ocean and Mediterranean Sea) for low predators (*n* = 65), mid predators (*n* = 417) and top predators (*n* = 276) or for all fish taken together (*n* = 795). A difference was only found for herbivore fish (*n* = 37). We therefore assigned Hg concentrations to all ISSCAAP without including a spatial component. The meta-analysis from Bonito and colleagues^50^ showed a decline in Hg concentrations in fish between 1969 and 2012 of 0.001 *μ*g g^−1^ a^−1^ (converted from a slope of −0.01 ng g^−1^ a^−1^ on a log scale; *R*^2^ = 0.03, *n* = 2,662). Over a period of 64 years in our study, this indicates that mean fish Hg concentrations were approximately 0.062 *μ*g g^−1^ higher in 1950 compared to 2014. However, we warrant caution on the interpretation of this trend given the low effect size of the published equation (*R*^2^ = 0.03) and consequently the uncertain predictive value. The Hg export estimates presented in our study were therefore not corrected for this potential time trend and are accurate for recent years but could be an underestimate for earlier years.

We tested for a significant change in slope in the time series of exported Hg and for the occurrence of a breakpoint (i.e., change in trend) using segmented regressions with the package *segmented*^51,52^ using the software R^53^. The Davies test was used to test for a non-zero difference-in-slope parameter of the segmented regression relationship^54^. Regression tests were two-tailed.

### Seafood consumption by country

Risk of Hg intake is to MeHg, which is the bioaccumulative and toxic form of Hg. We evaluated potential mean MeHg *per capita* weekly intake (WI) of marine fish and seafood for each country in this study (*μ*g kg of body mass (BM)^−1^ week^−1^) between 1961 and 2011 using the equation:

3 $$W{I}_{j}={{\rm{\Sigma }}}_{ij}(I{R}_{ij}\times {C}_{i})/B{M}_{k}/52$$

where *WI*_*j*_ is the potential *per capita* weekly intake of country *j*, *IR*_*ij*_ is the intake rate (kg capita^−1^ year^−1^) of a specific taxonomic group *i per capita* for country *j*, *C*_*i*_ is the mean MeHg concentration of taxonomic group *i*, and *BM*_*k*_ is the average human body mass for continent *k*. *IR*_*ij*_ was estimated using data for marine fish and seafood supply which was available for consumption (kg capita^−1^ year^−1^) from the FAO^22^ for each country from 1961 to 2011 (years when commodity balance sheets were complete). There are currently 193 United Nations member states, but fish and seafood supply data were not available for all countries during the studied period. In 1961, data were only available for 139 countries, however the number of reporting nations gradually increased to 174 in 2011. The available food supply is calculated by the FAO by adding importations to production (i.e. catches and aquaculture) and by removing exportations, non-food uses, and waste^22^. *Per capita* food supply was used here as a proxy for *per capita* food consumption^55^ and the WI calculated here are therefore estimates of the potential intake (food available *per capita* for each country) rather than the actual intake (food consumed *per capita* for each country); for this reason, we use the term “potential” WI. *C*_*i*_ was estimated using the weighted mean of MeHg (MeHg_w_; Supplementary Table 6), for each available marine taxonomic group. Over 95% of THg in edible fish parts (e.g., muscle) was assumed to be in the MeHg form^45,56^. MeHg_w_ of each available marine taxonomic group (“Cephalopods”, “Crustaceans”, “Demersal Fish”, “Other Marine Fish”, “Other Molluscs”, and “Pelagic Fish”; Supplementary Table 6) was multiplied by *IR*_*ij*_ of that group to estimate the WI for each country. A different average BM of human population was used for each continent: Asia = 57.7 kg, Africa = 60.7 kg, Latin America and the Caribbean = 67.9 kg, Europe = 70.8 kg, Oceania = 74.1 kg, and North America = 80.7 kg^57^. We compared the calculated WI to the provisional tolerable weekly intake (PTWI) of 1.6 *μ*g kg^−1^ week^−1^ for methylmercury^24^. We reported average WI (±SD) for the periods 1961–1970, 1971–1980, 1981–1990, 1991–2000, and 2001–2011.

### Data availability

The authors declare that the data supporting the findings of this study are available within the article and the Supplementary Information. Time series fishery data and available food supply are available at the FAO website (http://www.fao.org). The Hg database used to pair fisheries data to taxa-specific Hg concentrations can be found in Supplementary Table 3 and at the author’s website (http://www.stonybrook.edu/commcms/gelfond/fish/database.html)^34^. Additional data supporting the findings of this study are available from the corresponding author upon reasonable request.

## Electronic supplementary material

Supplementary Information

Supplementary Table S4
