# Supplementary material for: Mercury transport and human exposure from global marine fisheries
Source: Sci Rep. 2018 Apr 30;8:6705. doi: 10.1038/s41598-018-24938-3 (PMC5928114; doi:10.1038/s41598-018-24938-3)
Supplement: Supplementary file 1 — Supplementary Information [file 41598_2018_24938_MOESM1_ESM.docx]

Supplementary Information

**Mercury transport and human exposure from global marine fisheries**

Raphael A. Lavoie*, Ariane Bouffard, Roxane Maranger, and Marc Amyot

GRIL, Département de Sciences Biologiques, Université de Montréal, C.P. 6128, Succursale Centre-ville, Pavillon Marie-Victorin, Montréal, Québec H3C 3J7, Canada

Corresponding author e-mail: [lavoie.raphael@gmail.com](mailto:lavoie.raphael@gmail.com)

**Supplementary Table 1 | Results of segmented regressions with breakpoint year ± standard error (SE) and segmented line slopes with lower and upper 95% confidence intervals (CI) for fisheries catch time series of the entire ocean, the coastal ecosystem and the high seas presented in Fig. 1. The Davies test is testing for a non-zero difference-in-slope parameter.**

| **Catch time series** | **Segmented regression** | | | | **Davies test** | **Overall test** | |
| --- | --- | --- | --- | --- | --- | --- | --- |
|  | **Breakpoint (year ± SE)** | **Line** | **Slope (kmol a^-1^)** | **95% CI** |  | **R^2^_adj_** | **P** |
| Entire ocean |  | 1 | 1.25 | 1.21–1.29 |  | 0.992 | <0.001 |
|  | 1991 ± 0.7 | 2 | 0.04 | -0.05–0.14 | <0.001 |  |  |
| Coastal |  | 1 | 1.11 | 1.07–1.15 |  | 0.987 | <0.001 |
|  | 1989 ± 0.6 | 2 | -0.19 | -0.28– -0.10 | <0.001 |  |  |
| High seas |  | 1 | 0.10 | 0.08–0.13 |  | 0.988 | <0.001 |
|  | 1975 ± 1.3 | 2 | 0.27 | 0.26–0.28 | <0.001 |  |  |
|  |  |  |  |  |  |  |  |

**Supplementary Table 2 | Trophic level (TL; mean ± SE) from FishBase^1^ for each ISSCAAP group.**

| **ISSCAAP group** | **TL** | | | **Rounded TL ^a^** | **N** |
| --- | --- | --- | --- | --- | --- |
| Abalones, winkles, conchs | 2.1 | ± | 0.24 | 2 | 17 |
| Blue-whales, fin-whales | 3.8 | ± | 0.58 | 4 | 7 |
| Carps, barbels and other cyprinids | 2.8 | ± | 0.28 | 3 | 72 |
| Clams, cockles, arkshells | 2.1 | ± | 0.13 | 2 | 42 |
| Cods, hakes, haddocks | 3.9 | ± | 0.52 | 4 | 72 |
| Crabs, sea-spiders | 2.9 | ± | 0.38 | 3 | 25 |
| Eared seals, hair seals, walruses | 4.2 | ± | 0.59 | 4 | 18 |
| Flounders, halibuts, soles | 3.6 | ± | 0.42 | 4 | 59 |
| Freshwater crustaceans | 2.2 | ± | 0.36 | 2 | 19 |
| Freshwater molluscs | 2.1 | ± | 0.15 | 2 | 3 |
| Herrings, sardines, anchovies | 3.0 | ± | 0.27 | 3 | 55 |
| Horseshoe crabs and other arachnoids | 2.3 | ± | 0.61 | 2 | 1 |
| King crabs, squat-lobsters | 2.3 | ± | 0.24 | 2 | 14 |
| Krill, planktonic crustaceans | 3.1 | ± | 0.34 | 3 | 2 |
| Lobsters, spiny-rock lobsters | 2.7 | ± | 0.35 | 3 | 26 |
| Marine fishes not identified | 3.7 | ± | 0.46 | 4 | 1113 |
| Miscellaneous aquatic invertebrates | 2.7 | ± | 0.53 | 3 | 5 |
| Miscellaneous aquatic mammals | 2.8 | ± | 0.22 | 3 | 2 |
| Miscellaneous coastal fishes | 3.6 | ± | 0.43 | 4 | 420 |
| Miscellaneous demersal fishes | 3.8 | ± | 0.47 | 4 | 181 |
| Miscellaneous diadromous fishes | 3.8 | ± | 0.54 | 4 | 8 |
| Miscellaneous freshwater fishes | 3.4 | ± | 0.42 | 3 | 156 |
| Miscellaneous marine crustaceans | 2.6 | ± | 0.32 | 3 | 7 |
| Miscellaneous marine molluscs | 2.1 | ± | 0.13 | 2 | 1 |
| Miscellaneous pelagic fishes | 3.9 | ± | 0.48 | 4 | 124 |
| Mussels | 2.1 | ± | 0.22 | 2 | 13 |
| Oysters | 2.1 | ± | 0.13 | 2 | 15 |
| River eels | 3.7 | ± | 0.50 | 4 | 5 |
| Salmons, trouts, smelts | 3.5 | ± | 0.35 | 4 | 38 |
| Scallops, pectens | 2.1 | ± | 0.29 | 2 | 15 |
| Sea-squirts and other tunicates | 3.0 | ± | 0.30 | 3 | 4 |
| Sea-urchins and other echinoderms | 2.3 | ± | 0.61 | 2 | 10 |
| Shads | 3.3 | ± | 0.37 | 3 | 25 |
| Sharks, rays, chimaeras | 4.0 | ± | 0.49 | 4 | 155 |
| Shrimps, prawns | 2.6 | ± | 0.35 | 3 | 64 |
| Sperm-whales, pilot-whales | 4.4 | ± | 0.53 | 4 | 27 |
| Squids, cuttlefishes, octopuses | 3.7 | ± | 0.50 | 4 | 30 |
| Sturgeons, paddlefishes | 3.3 | ± | 0.39 | 3 | 11 |
| Tilapias and other cichlids | 2.8 | ± | 0.25 | 3 | 25 |
| Tunas, bonitos, billfishes | 4.3 | ± | 0.66 | 4 | 47 |
| Turtles | 2.6 | ± | 0.33 | 3 | 7 |

^a^ Trophic level rounded to the nearest integer.

**Supplementary Table 3 | Total mercury (THg) and methylmercury (MeHg) mean concentrations (*μ*g g^-1^) ± standard deviation for each 41 ISSCAAP group weighted for samples sizes (Hg_w_ ± SD_w_). These concentrations were paired with annual catch of marine fisheries of the same ISSCAAP groups to estimate Hg export.**

| **ISSCAAP group** | **Measured Hg_w_ ^a^** | | | **Whole THg_w_ ^b^** | | | **Whole MeHg_w_ ^c^** | ***N*** | **Reference ^d^** |
| --- | --- | --- | --- | --- | --- | --- | --- | --- | --- |
| Abalones, winkles, conchs | 0.007 | ± | 0.007 | 0.007 | ± | 0.007 | 0.003 | 15 | ^2^ |
| Blue-whales, fin-whales | 0.243 | ± | 0.351 | 0.139 | ± | 0.199 | 0.026 | 262 | ^3-8^ |
| Carps, barbels and other cyprinids | 0.165 | ± | 0.093 | 0.105 | ± | 0.055 | 0.061 | 420 | ^9^ |
| Clams, cockles, arkshells | 0.024 | ± | 0.022 | 0.024 | ± | 0.022 | 0.011 | 816 | ^9,10^ |
| Cods, hakes, haddocks | 0.105 | ± | 0.076 | 0.068 | ± | 0.046 | 0.040 | 3062 | ^9^ |
| Crabs, sea-spiders | 0.102 | ± | 0.088 | 0.102 | ± | 0.088 | 0.047 | 1361 | ^9^ |
| Eared seals, hair seals, walruses | 13.1 | ± | 17.8 | 0.450 | ± | 0.601 | 0.086 | 1439 | ^4,5,10-24^ |
| Flounders, halibuts, soles | 0.190 | ± | 0.100 | 0.119 | ± | 0.060 | 0.069 | 6141 | ^9^ |
| Freshwater crustaceans | 0.210 |  |  | 0.210 |  |  | 0.097 | 1 | ^9^ |
| Freshwater molluscs | 0.020 | ± | 0.013 | 0.020 | ± | 0.013 | 0.009 | 95 | ^25,26^ |
| Herrings, sardines, anchovies | 0.069 | ± | 0.040 | 0.046 | ± | 0.025 | 0.027 | 2314 | ^9^ |
| Horseshoe crabs and other arachnoids | 0.077 | ± | 0.020 | 0.077 | ± | 0.020 | 0.036 | 214 | ^27,28^ |
| King crabs, squat-lobsters | 0.096 | ± | 0.085 | 0.096 | ± | 0.085 | 0.044 | 2102 | ^2,5,9-11,29^ |
| Krill, planktonic crustaceans | 0.013 | ± | 0.008 | 0.013 | ± | 0.008 | 0.006 | 61 | ^2,5,10,11,29^ |
| Lobsters, spiny-rock lobsters | 0.185 | ± | 0.071 | 0.185 | ± | 0.071 | 0.085 | 172 | ^9^ |
| Marine fishes not identified | 0.340 | ± | 0.454 | 0.202 | ± | 0.247 | 0.117 | 32480 | ^9,30,31^ |
| Miscellaneous aquatic invertebrates | 0.047 | ± | 0.061 | 0.047 | ± | 0.061 | 0.022 | 7530 | ^2,5,9-11,25-29^ |
| Miscellaneous aquatic mammals | 10.9 | ± | 37.0 | 1.74 | ± | 7.7 | 0.331 | 2346 | ^3-8,10-24,32-41^ |
| Miscellaneous coastal fishes | 0.264 | ± | 0.194 | 0.162 | ± | 0.113 | 0.094 | 5212 | ^9^ |
| Miscellaneous demersal fishes | 0.169 | ± | 0.140 | 0.106 | ± | 0.081 | 0.062 | 10126 | ^9^ |
| Miscellaneous diadromous fishes | 0.139 | ± | 0.125 | 0.088 | ± | 0.075 | 0.051 | 4586 | ^9,30,31^ |
| Miscellaneous freshwater fishes | 0.227 | ± | 0.146 | 0.141 | ± | 0.086 | 0.082 | 9840 | ^9^ |
| Miscellaneous marine crustaceans | 0.096 | ± | 0.085 | 0.096 | ± | 0.085 | 0.044 | 2102 | ^2,5,9-11,29^ |
| Miscellaneous marine molluscs | 0.020 | ± | 0.019 | 0.020 | ± | 0.019 | 0.009 | 4368 | ^2,9,10^ |
| Miscellaneous pelagic fishes | 0.587 | ± | 0.637 | 0.341 | ± | 0.341 | 0.198 | 12257 | ^9^ |
| Mussels | 0.026 | ± | 0.015 | 0.026 | ± | 0.015 | 0.012 | 589 | ^9^ |
| Oysters | 0.015 | ± | 0.014 | 0.015 | ± | 0.014 | 0.007 | 2815 | ^9^ |
| River eels | 0.220 | ± | 0.118 | 0.138 | ± | 0.068 | 0.080 | 535 | ^9^ |
| Salmons, trouts, smelts | 0.050 | ± | 0.015 | 0.034 | ± | 0.010 | 0.020 | 2204 | ^9^ |
| Scallops, pectens | 0.064 | ± | 0.027 | 0.064 | ± | 0.027 | 0.030 | 133 | ^9^ |
| Sea-squirts and other tunicates | 0.340 | ± | 0.454 | 0.202 | ± | 0.247 | 0.093 | 32480 | ^9,30,31^ |
| Sea-urchins and other echinoderms | 0.009 | ± | 0.011 | 0.009 | ± | 0.011 | 0.004 | 15 | ^2,10^ |
| Shads | 0.077 | ± | 0.029 | 0.051 | ± | 0.018 | 0.030 | 93 | ^9^ |
| Sharks, rays, chimaeras | 0.893 | ± | 0.487 | 0.515 | ± | 0.257 | 0.299 | 3021 | ^9^ |
| Shrimps, prawns | 0.058 | ± | 0.046 | 0.058 | ± | 0.046 | 0.027 | 508 | ^2,5,9^ |
| Sperm-whales, pilot-whales | 10.2 | ± | 65.0 | 5.27 | ± | 14.1 | 1.00 | 645 | ^4,7,10,32-41^ |
| Squids, cuttlefishes, octopuses | 0.061 | ± | 0.055 | 0.061 | ± | 0.055 | 0.028 | 735 | ^9^ |
| Sturgeons, paddlefishes | 0.139 | ± | 0.106 | 0.088 | ± | 0.064 | 0.051 | 157 | ^30,31^ |
| Tilapias and other cichlids | 0.196 | ± | 0.128 | 0.123 | ± | 0.075 | 0.071 | 2907 | ^9^ |
| Tunas, bonitos, billfishes | 0.665 | ± | 0.733 | 0.384 | ± | 0.385 | 0.223 | 6051 | ^9^ |
| Turtles | 0.028 | ± | 0.014 | 0.028 | ± | 0.014 | 0.011 | 28 | ^42,43^ |

^a^ Measurements made on edible portion (fillet or whole for small fish)^9^. Hg measurements on edible portions are assumed to be representative of THg and MeHg^9^.

^b^ THg concentration in fish muscle for each ISSCAAP group was transformed into whole body concentrations according to the equation (1) of main text^44^.

^c^ We used percent of THg as MeHg of 58% for whole fish (n = 39), 46% for whole invertebrates (n = 462), and 39% for various tissues of turtles (n = 944). For marine mammals, we estimated the whole body burden of MeHg using published %MeHg resulting in an average of 19% of THg as MeHg (n = 206).

^d^ The Hg Seafood Database^9^ can be found at the author’s website (<http://www.stonybrook.edu/commcms/gelfond/fish/database.html>).

**Supplementary Table 4 | Per capita methylmercury (MeHg) weekly intake (WI; μg of MeHg / kg of body mass (BM) / week) of marine fish and seafood between 1961 and 2011 for each country based on data of fish and seafood supply available for food consumption from the FAO.**

See spreadsheet “Lavoie et al_Hg Fisheries_Supplementary Table4.xlsx”.

**Supplementary Table 5 | Average mass (mean ± SD, in metric tonnes; t) of odontocetes, mysticetes and pinnipeds.**

| **Common name** | **Scientific name** | **Mass (mean ± SD; t)** | | | ***n*** | **Reference** |
| --- | --- | --- | --- | --- | --- | --- |
| **Odontocetes ("Sperm-whales, pilot-whales" group)** | |  |  |  |  |  |
| Commerson's Dolphin | *Cephalorhynchus commersonii* | 0.070 | ± | 0.009 | 11 | ^45^ |
| Heaviside's Dolphin | *Cephalorhynchus heavisidii* | 0.071 |  |  | 1 | ^45^ |
| Beluga Whale | *Delphinapterus leucas* | 0.38 | ± | 0.07 | 3 | ^46^ |
| Short-beaked Common Dolphin | *Delphinus delphis* | 0.088 | ± | 0.030 | 246 | ^45^ |
| Pygmy Killer Whale | *Feresa attenuata* | 0.11 | ± | 0.01 | 9 | ^45^ |
| Short-finned Pilot Whale | *Globicephala macrorhynchus* | 0.93 | ± | 0.44 | 9 | ^45^ |
| Long-finned Pilot Whale | *Globicephala melas* | 0.70 | ± | 0.49 | 19 | ^45^ |
| Risso's Dolphin | *Grampus griseus* | 0.28 | ± | 0.09 | 18 | ^45^ |
| Boto | *Inia geoffrensis* | 0.08 | ± | 0.03 | 4 | ^45^ |
| Pygmy Sperm Whale | *Kogia breviceps* | 0.35 | ± | 0.13 | 47 | ^45^ |
| Dwarf Sperm Whale | *Kogia sima* | 0.15 | ± | 0.05 | 20 | ^45^ |
| Fraser's Dolphin | *Lagenodelphis hosei* | 0.23 | ± | 0.13 | 2 | ^45^ |
| Atlantic White-sided Dolphin | *Lagenorhynchus acutus* | 0.15 | ± | 0.04 | 66 | ^45^ |
| White-beaked Dolphin | *Lagenorhynchus albirostris* | 0.22 | ± | 0.03 | 3 | ^45^ |
| Pacific White-sided Dolphin | *Lagenorhynchus obliquidens* | 0.11 | ± | 0.03 | 14 | ^45^ |
| Northern Right Whale Dolphin | *Lissodelphis borealis* | 0.077 | ± | 0.013 | 5 | ^45^ |
| Sowerby's Beaked Whale | *Mesoplodon bidens* | 0.65 |  |  | 1 | ^45^ |
| Hubbs' Beaked Whale | *Mesoplodon carlhubbsi* | 0.99 | ± | 0.62 | 2 | ^45^ |
| Blainville's Beaked Whale | *Mesoplodon densirostris* | 0.78 | ± | 0.23 | 6 | ^45^ |
| Gervais' Beaked Whale | *Mesoplodon europaeus* | 0.70 | ± | 0.15 | 13 | ^45^ |
| Ginkgo-toothed Beaked Whale | *Mesoplodon ginkgodens* | 1.0 | ± | 0.41 | 3 | ^45^ |
| Gray's Beaked Whale | *Mesoplodon grayi* | 1.0 |  |  | 1 | ^45^ |
| True's Beaked Whale | *Mesoplodon mirus* | 1.1 | ± | 0.23 | 3 | ^45^ |
| Pygmy Beaked Whale | *Mesoplodon peruvianus* | 0.28 |  |  | 1 | ^45^ |
| Indo-Pacific Finless Porpoise | *Neophocaena phocaenoides* | 0.033 |  |  | 1 | ^45^ |
| Irrawaddy Dolphin | *Orcaella brevirostris* | 0.084 | ± | 0.00 | 2 | ^45^ |
| Killer Whale | *Orcinus orca* | 1.8 | ± | 1.1 | 40 | ^47^ |
| Melon-headed Whale | *Peponocephala electra* | 0.17 | ± | 0.04 | 3 | ^45^ |
| Porpoise | *Phocaena phocaena* | 0.049 | ± | 0.015 | 96 | ^45,46^ |
| Vaquita | *Phocoena sinus* | 0.045 | ± | 0.006 | 4 | ^45^ |
| Dall's Porpoise | *Phocoenoides dalli* | 0.10 | ± | 0.03 | 35 | ^45^ |
| Dall's Porpoise | *Phocoenoides truei* | 0.10 | ± | 0.01 | 2 | ^45^ |
| Sperm Whale | *Physeter macrocephalus* | 31 | ± | 12 | 50 | ^45,48^ |
| Ganges River Dolphin | *Platanista gangetica* | 0.066 | ± | 0.013 | 7 | ^45^ |
| Franciscana | *Pontoporia blainvillei* | 0.031 | ± | 0.008 | 30 | ^45^ |
| False Killer Whale | *Pseudorca crassidens* | 0.54 | ± | 0.20 | 7 | ^45^ |
| Tucuxi | *Sotalia fluviatilis* | 0.030 |  |  | 1 | ^45^ |
| Pantropical Spotted Dolphin | *Stenella attenuata* | 0.061 | ± | 0.015 | 125 | ^45^ |
| Clymene Dolphin | *Stenella clymene* | 0.066 | ± | 0.028 | 2 | ^45^ |
| Striped Dolphin | *Stenella coeruleoalba* | 0.093 | ± | 0.036 | 164 | ^45^ |
| Atlantic Spotted Dolphin | *Stenella frontalis* | 0.10 | ± | 0.02 | 25 | ^45^ |
| Spinner Dolphin | *Stenella longirostris* | 0.062 | ± | 0.008 | 29 | ^45^ |
| Rough-toothed Dolphin | *Steno bredanensis* | 0.11 | ± | 0.02 | 25 | ^45^ |
| Shepherd's Beaked Whale | *Tasmacetus shepherdi* | 2.1 |  |  | 1 | ^45^ |
| Indo-Pacific Bottlenose Dolphin | *Tursiops aduncus* | 0.18 | ± | 0.02 | 4 | ^45^ |
| Common Bottlenose Dolphin | *Tursiops truncatus* | 0.21 | ± | 0.06 | 275 | ^45^ |
| Cuvier's Beaked Whale | *Ziphius cavirostris* | 1.6 | ± | 0.86 | 14 | ^45^ |
| Mean odontocetes |  | 1.3 | ± | 6.1 | 1449 |  |
| **Mysticetes ("Blue-whales, fin-whales" group)** | |  |  |  |  |  |
| Minke Whale | *Balaenoptera acutorostrata* | 5.8 | ± | 2.4 | 23 | ^48^ |
| Sei Whale | *Balaenoptera borealis* | 14 | ± | 6.7 | 20 | ^48^ |
| Bryde Whale | *Balaenoptera brydei* | 13 | ± | 2.1 | 27 | ^48^ |
| Blue Whale | *Balaenoptera musculus* | 88 | ± | 34 | 44 | ^48^ |
| Blue Whale | *Balaenoptera musculus* | 58 |  |  | 1 | ^46^ |
| Pigmy blue Whale | *Balaenoptera musculus brevicauda* | 47 | ± | 17 | 5 | ^48^ |
| Fin Whale | *Balaenoptera physalus* | 49 | ± | 9.3 | 42 | ^48^ |
| Gray Whale | *Eschrichtius robustus* | 17 | ± | 10 | 8.0 | ^48^ |
| Pacific right Whale | *Eubalaena glacialis sieboldii* | 58 | ± | 20 | 18 | ^48^ |
| Humpback Whale | *Megaptera novaeangliae* | 34 | ± | 6.0 | 7 | ^48^ |
| Mean mysticetes |  | 43 | ± | 35 | 195 |  |
| **Pinnipeds ("Eared seals, hair seals, walruses" group)** | |  |  |  |  |  |
| Guadalupe fur seal | *Arctocephalus townsendi* | 0.049 | ± | 0.0057 | 14 | ^49^ |
| Bearded seal | *Erignathus barbatus* | 0.20 | ± | 0.12 | 2 | ^46^ |
| Grey seal | *Halichoevus gvypus* | 0.26 | ± | 0.026 | 23 | ^50^ |
| Weddell seal | *Leptonychotes weddellii* | 0.35 | ± | 0.047 | 2 | ^12^ |
| Elephant seal | *Mirounga sp.* | 2.0 |  |  | 1 | ^51^ |
| Walrus | *Odobenus rosmaru* | 0.35 | ± | 0.33 | 4 | ^46^ |
| Ringed seal | *Phoca hispida* | 0.040 | ± | 0.0002 | 2 | ^46^ |
| Seal | *Phoca richardi geronimensis* | 0.11 |  |  | 1 | ^46^ |
| Mean pinnipeds |  | 0.23 | ± | 0.29 | 49 |  |

**Supplementary Table 6 | Methylmercury (MeHg) mean concentrations (*μ*g g^-1^) ± standard deviation for each taxonomic group weighted for samples sizes (Hg_w_ ± SD_w_).**

| **Taxonomic group ^a^** | **MeHg_w_ ^b^** | | | ***N*** | **Reference ^c^** |
| --- | --- | --- | --- | --- | --- |
| Crustaceans | 0.096 | ± | 0.085 | 2102 | ^2,5,9-11,29^ |
| Cephalopods | 0.047 | ± | 0.061 | 7530 | ^2,5,9-11,25-29^ |
| Demersal fish | 0.169 | ± | 0.140 | 10126 | ^9^ |
| Molluscs, Other | 0.020 | ± | 0.019 | 4368 | ^2,9,10^ |
| Pelagic fish | 0.587 | ± | 0.637 | 12257 | ^9^ |
| Marine fish, Other | 0.340 | ± | 0.454 | 32480 | ^9,30,31^ |

^a^ Taxonomic groups for which available food supply data (kg capita^-1^ year^-1^) are accessible from the FAO^52^.

^b^ Measurements made on edible portion (fillet or whole for small fish)^9^. Hg measurements on edible portions are assumed to be representative of THg and MeHg^9^.

^c^ The Hg Seafood Database^9^ can be found at the author’s website (<http://www.stonybrook.edu/commcms/gelfond/fish/database.html>).

**Supplementary Table 7 | List of Major Fishing Areas (MFA) for statistical purposes**

| MFA name | MFA number |
| --- | --- |
| Arctic Sea | 18 |
| Atlantic, Northwest | 21 |
| Atlantic, Northeast | 27 |
| Atlantic, Western Central | 31 |
| Atlantic, Eastern Central | 34 |
| Mediterranean and Black Sea | 37 |
| Atlantic, Southwest | 41 |
| Atlantic, Southeast | 47 |
| Atlantic, Antarctic | 48 |
| Indian Ocean, Western | 51 |
| Indian Ocean, Eastern | 57 |
| Indian Ocean, Antarctic | 58 |
| Pacific, Northwest | 61 |
| Pacific, Northeast | 67 |
| Pacific, Western Central | 71 |
| Pacific, Eastern Central | 77 |
| Pacific, Southwest | 81 |
| Pacific, Southeast | 87 |
| Pacific, Antarctic | 88 |

**
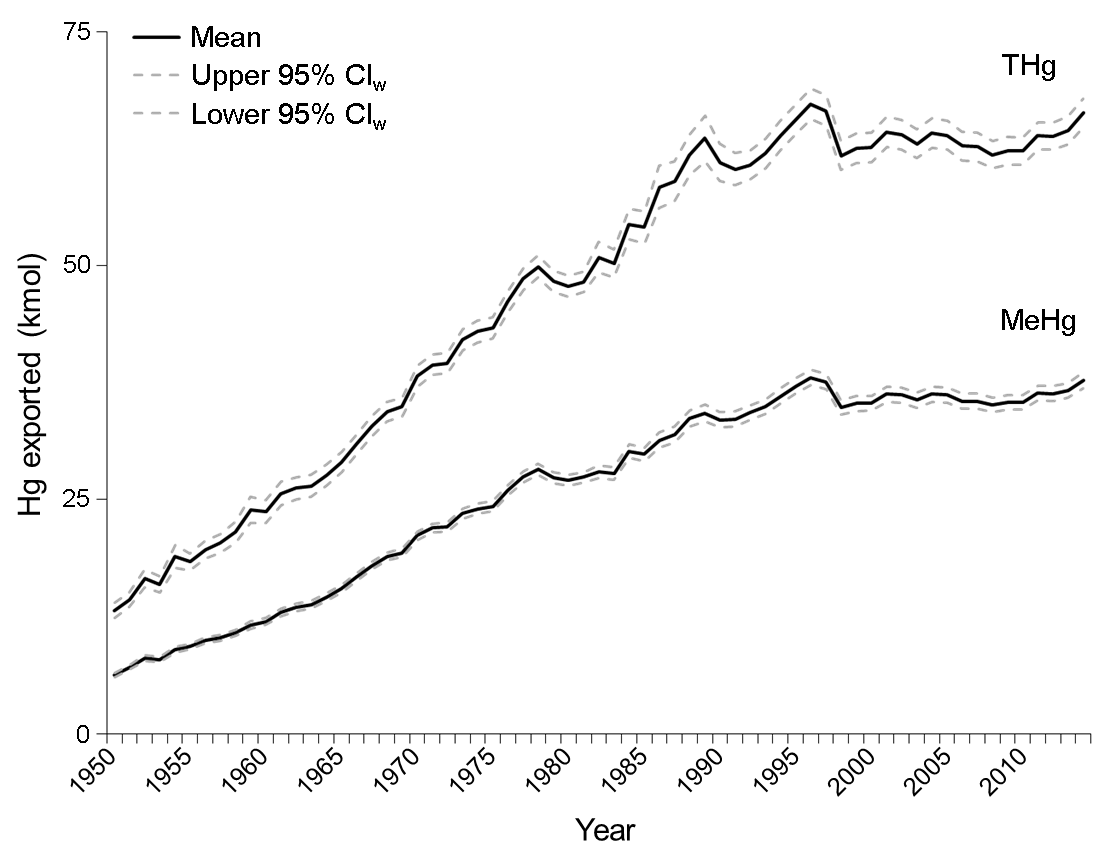
Supplementary Figure 1 | Temporal trends (1950‒2014) of Hg exported from the entire ocean resulting from marine fisheries.** Average (full line) with lower and upper 95% confidence intervals weighted for sample size (CI_w_; dashed lines) are shown.


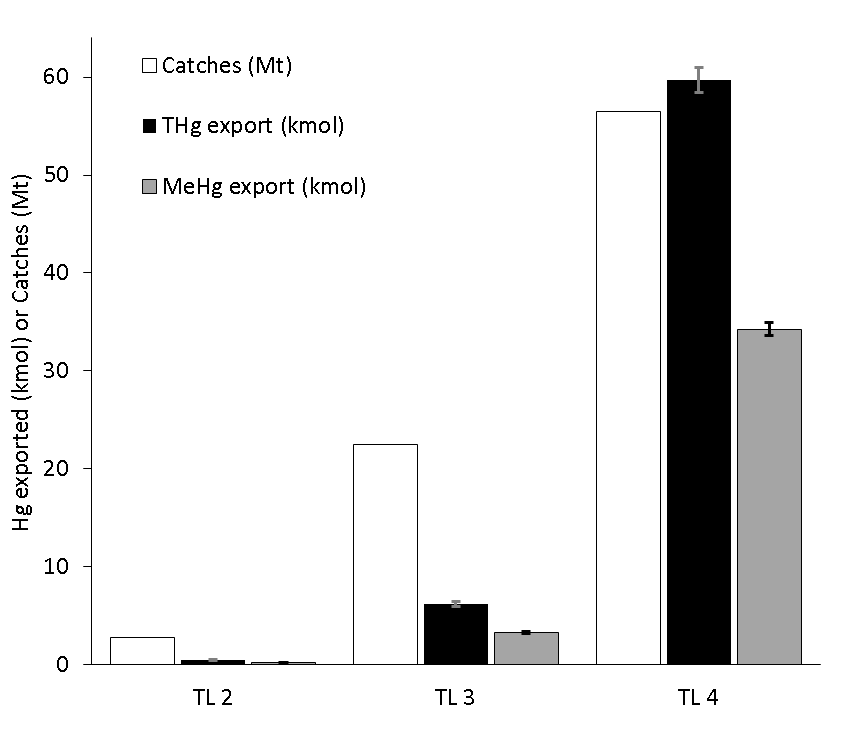


**Supplementary Figure 2 | Catches (white) and total mercury (THg; black) and methylmercury (MeHg; grey) exported from the entire ocean resulting from marine fisheries in 2014 by trophic level (TL).** Averages with lower and upper 95% confidence intervals weighted for sample size (CI_w_; error bars) are shown for Hg export.


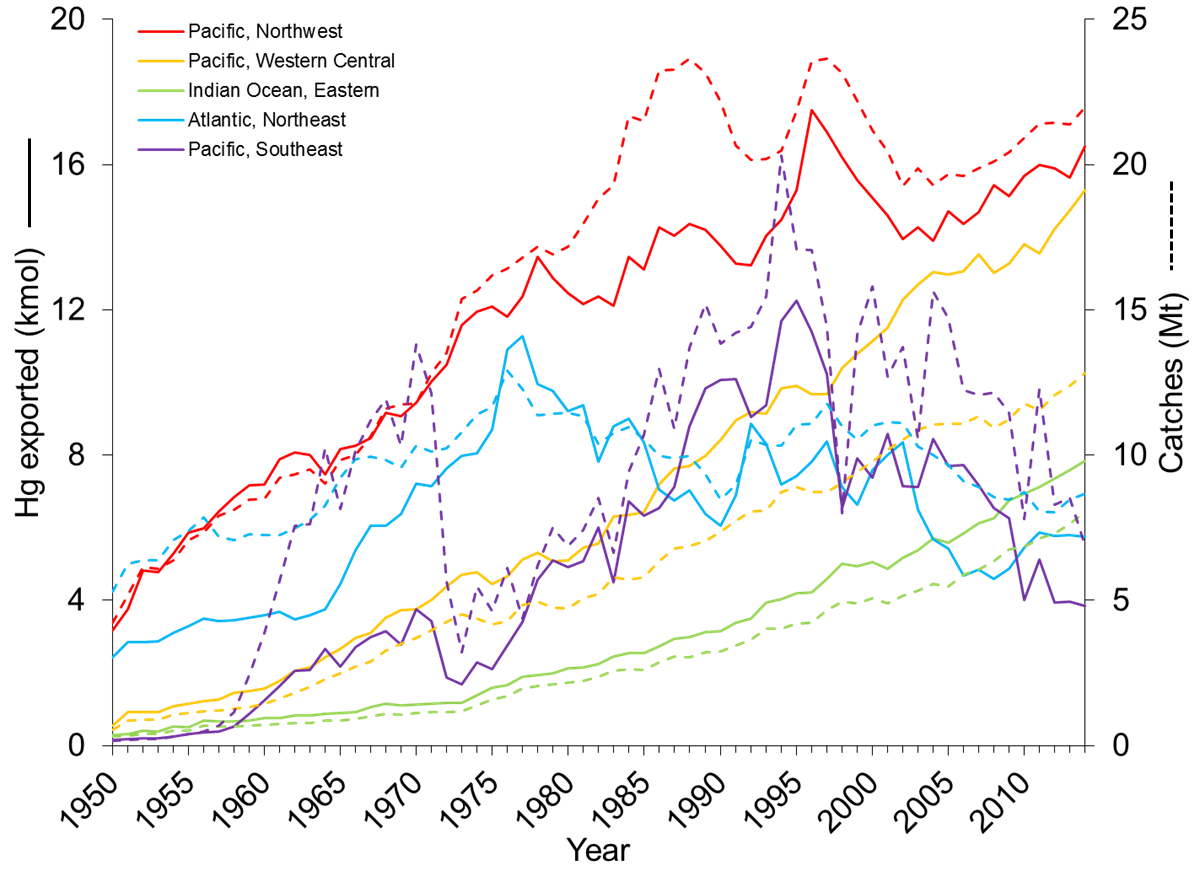


**Supplementary Figure 3 | Temporal trends (1950‒2014) of catches (dashed lines) and Hg exported (full lines).** Northwest Pacific (#61; red), Western Central Pacific (#71; orange), Eastern Indian Ocean (#57; green), Northeast Atlantic (#27; blue), and Southeast Pacific (#87; purple).

**
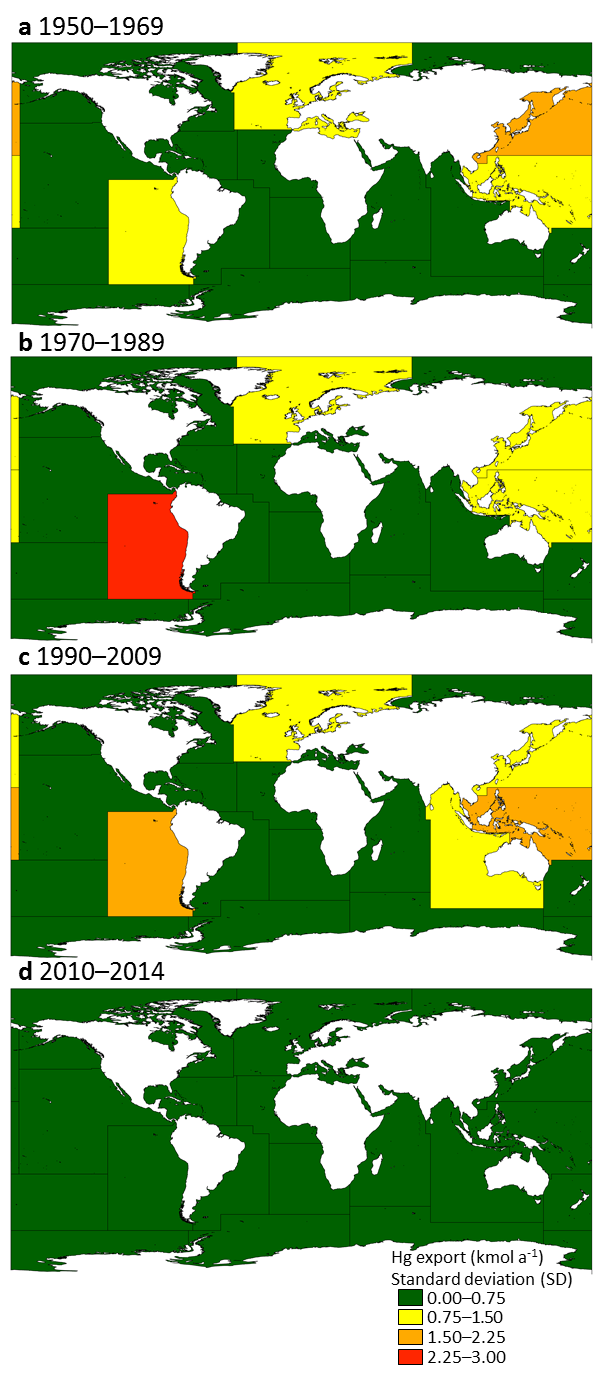
**

**Supplementary Figure 4 | Hg export from the ocean.** Standard deviations of mean total Hg export data in **a**, 1950–1969, **b**, 1970–1989, **c**, 1990–2009, and **d**, 2010–2014 resulting from catches of fishes, molluscs, crustaceans, reptiles, and mammals for each Major Fishing Areas (MFA). MFA numbers are provided in panel **a** and corresponding MFA names are found in Supplementary Table 7. Means for each period are found in Fig. 2. Maps were created in ArcGIS version 10.3.1 (<http://desktop.arcgis.com/>).

**References**

1 Froese, R. & Pauly, D. FishBase. http://fishbase.org. (2016).

2 Lavoie, R. A. *et al.* Trophic structure and mercury distribution in a Gulf of St. Lawrence (Canada) food web using stable isotope analysis. *Sci. Total Environ.* **408**, 5529-5539, doi:10.1016/j.scitotenv.2010.07.053 (2010).

3 Bilandzic, N. *et al.* Concentration of mercury and selenium in tissues of five cetacean species from Croatian coastal waters. *Arch. Biol. Sci.* **67**, 1377-1389, doi:10.2298/abs140215116b (2015).

4 Dehn, L. A. *et al.* Trophic relationships in an Arctic food web and implications for trace metal transfer. *Sci. Total Environ.* **362**, 103-123, doi:10.1016/j.scitotenv.2005.11.012 (2006).

5 Riget, F. *et al.* Transfer of mercury in the marine food web of West Greenland. *J. Environ. Monit.* **9**, 877-883, doi:10.1039/b704796g (2007).

6 Born, E. W. *et al.* Population substructure of North Atlantic minke whales (Balaenoptera acutorostrata) inferred from regional variation of elemental and stable isotopic signatures in tissues. *J. Mar. Syst.* **43**, 1-17, doi:10.1016/s0924-7963(03)00085-x (2003).

7 Hansen, A. M. K., Bryan, C. E., West, K. & Jensen, B. A. Trace Element Concentrations in Liver of 16 Species of Cetaceans Stranded on Pacific Islands from 1997 through 2013. *Arch. Environ. Contam. Toxicol.* **70**, 75-95, doi:10.1007/s00244-015-0204-1 (2016).

8 Tilbury, K. L. *et al.* Chemical contaminants in juvenile gray whales (Eschrichtius robustus) from a subsistence harvest in Arctic feeding grounds. *Chemosphere* **47**, 555-564, doi:10.1016/s0045-6535(02)00061-9 (2002).

9 Karimi, R., Fitzgerald, T. P. & Fisher, N. S. A quantitative synthesis of mercury in commercial seafood and implications for exposure in the United States. *Environ. Health Perspect.* **120**, 1512-1519, doi:10.1289/ehp.1205122 (2012).

10 Atwell, L., Hobson, K. A. & Welch, H. E. Biomagnification and bioaccumulation of mercury in an Arctic marine food web: insights from stable nitrogen isotope analysis. *Can. J. Fish. Aquat. Sci.* **55**, 1114-1121, doi:10.1139/f98-001 (1998).

11 Jarman, W. M., Hobson, K. A., Sydeman, W. J., Bacon, C. E. & McLaren, E. B. Influence of trophic position and feeding location on contaminant levels in the Gulf of the Farallones food web revealed by stable isotope analysis. *Environ. Sci. Technol.* **30**, 654-660, doi:10.1021/es950392n (1996).

12 Yamamoto, Y., Honda, K., Hidaka, H. & Tatsukawa, R. Tissue distribution of heavy-metals in Weddell Seals (*Leptonychotes weddellii*). *Mar. Pollut. Bull.* **18**, 164-169, doi:10.1016/0025-326x(87)90240-2 (1987).

13 Wagemann, R., Innes, S. & Richard, P. R. Overview and regional and temporal differences of heavy metals in Arctic whales and ringed seals in the Canadian Arctic. *Sci. Total Environ.* **186**, 41-66, doi:10.1016/0048-9697(96)05085-1 (1996).

14 Warburton, J. & Seagars, D. J. *Metal concentrations in liver and kidney tissues of Pacific walrus: continuation of a baseline study*. (Marine Mammals Management, U.S. Fish and Wildlife Service, 1993).

15 Bustamante, P., Morales, C. F., Mikkelsen, B., Dam, M. & Caurant, F. Trace element bioaccumulation in grey seals Halichoerus grypus from the Faroe Islands. *Mar. Ecol. Prog. Ser.* **267**, 291-301, doi:10.3354/meps267291 (2004).

16 Dietz, R., Riget, F. & Johansen, P. Lead, cadmium, mercury and selenium in Greenland marine animals. *Sci. Total Environ.* **186**, 67-93, doi:10.1016/0048-9697(96)05086-3 (1996).

17 Julshamn, K. & Grahl-Nielsen, O. Trace element levels in harp seal (*Pagophilus groenlandicus*) and hooded seal (*Cystophora cristata*) from the Greenland Sea. A multivariate approach. *Sci. Total Environ.* **250**, 123-133, doi:10.1016/s0048-9697(00)00371-5 (2000).

18 Kretzmann, M., Rohrbach, L., Durham, K., DiGiovanni, R. & Sanudo-Wilhelmy, S. Trace metal burdens in stranded seals from Long Island, New York: potential evidence for species differences in foraging. *Aquat. Mamm.* **36**, 178-187, doi:10.1578/am.36.2.2010.178 (2010).

19 Medvedev, N., Panichev, N. & Hyvarinen, H. Levels of heavy metals in seals of Lake Ladoga and the White Sea. *Sci. Total Environ.* **206**, 95-105, doi:10.1016/S0048-9697(97)80001-0 (1997).

20 Miles, A. K., Calkins, D. G. & Coon, N. C. Toxic elements and organochlorines in harbor seals (*Phoca vitulina richardsi*), Kodiak, Alaska, USA. *Bull. Environ. Contam. Toxicol.* **48**, 727-732 (1992).

21 Smith, T. G. & Armstrong, F. A. J. Mercury and selenium in ringed and bearded seal tissues from Arctic Canada. *Arctic* **31**, 75-84 (1978).

22 Taylor, D. L., Schliebe, S. & Metsker, H. Contaminants in blubber, liver and kidney tissue of Pacific walruses. *Mar. Pollut. Bull.* **20**, 465-468, doi:10.1016/0025-326x(89)90069-6 (1989).

23 Wagemann, R. & Stewart, R. E. A. Concentrations of heavy metals and selenium in tissues and some foods of Walrus (*Odobenus rosmarus rosmarus*) from the eastern Canadian Arctic and Sub-Arctic, and associations between metals, age, and gender. *Can. J. Fish. Aquat. Sci.* **51**, 426-436, doi:10.1139/f94-044 (1994).

24 Wagemann, R., Stewart, R. E. A., Lockhart, W. L., Stewart, B. E. & Povoledo, M. Trace metals and methyl mercury: associations and transfer in harp seal (*Phoca groenlandica*) mothers and their pups. *Mar. Mamm. Sci.* **4**, 339-355, doi:10.1111/j.1748-7692.1988.tb00542.x (1988).

25 Fliedner, A. *et al.* Spatial and temporal trends of metals and arsenic in German freshwater compartments. *Environ. Sci. Pollut. Res.* **21**, 5521-5536, doi:10.1007/s11356-013-2487-y (2014).

26 Kidd, K. A. *et al.* Biomagnification of mercury through lake trout (*Salvelinus namaycush*) food webs of lakes with different physical, chemical and biological characteristics. *Sci. Total Environ.* **438**, 135-143, doi:10.1016/j.scitotenv.2012.08.057 (2012).

27 Burger, J., Dixon, C., Shukla, T., Tsipoura, N. & Gochfeld, M. Metal levels in horseshoe crabs (*Limulus polyphemus*) from Maine to Florida. *Environ. Res.* **90**, 227-236, doi:10.1016/s0013-9351(02)00027-0 (2002).

28 Burger, J. *et al.* Metals in horseshoe crabs from Delaware Bay. *Arch. Environ. Contam. Toxicol.* **44**, 36-42, doi:10.1007/s00244-002-2002-9 (2003).

29 Al-Reasi, H. A., Ababneh, F. A. & Lean, D. R. Evaluating mercury biomagnification in fish from a tropical marine environment using stable isotopes (delta C-13 and delta N-15). *Environ. Toxicol. Chem.* **26**, 1572-1581, doi:10.1897/06-359R.1 (2007).

30 Agusa, T., Kunito, T., Tanabe, S., Pourkazemi, M. & Aubrey, D. G. Concentrations of trace elements in muscle of sturgeons in the Caspian Sea. *Mar. Pollut. Bull.* **49**, 789-800, doi:10.1016/j.marpolbul.2004.06.008 (2004).

31 Greenfield, B. K. *et al.* Seasonal, interannual, and long-term variation in sport fish contamination, San Francisco Bay. *Sci. Total Environ.* **336**, 25-43, doi:10.1016/j.scitotenv.2004.05.023 (2005).

32 Becker, P. R. Concentration of chlorinated hydrocarbons and heavy metals in Alaska Arctic marine mammals. *Mar. Pollut. Bull.* **40**, 819-829, doi:10.1016/s0025-326x(00)00076-x (2000).

33 Becker, P. R. *et al.* Relationship of silver with selenium and mercury in the liver of two species of toothed whales (odontocetes). *Mar. Pollut. Bull.* **30**, 262-271, doi:10.1016/0025-326x(94)00176-a (1995).

34 Ferreira, M. *et al.* Biological variables and health status affecting inorganic element concentrations in harbour porpoises (*Phocoena phocoena*) from Portugal (western Iberian Peninsula). *Environ. Pollut.* **210**, 293-302, doi:10.1016/j.envpol.2016.01.027 (2016).

35 Frodello, J. P., Romeo, M. & Viale, D. Distribution of mercury in the organs and tissues of five toothed-whale species of the Mediterranean. *Environ. Pollut.* **108**, 447-452, doi:10.1016/s0269-7491(99)00221-3 (2000).

36 Hansen, C. T., Nielsen, C. O., Dietz, R. & Hansen, M. M. Zinc, cadmium, mercury and selenium in minke whales, belugas and narwhals from West Greenland. *Polar Biol.* **10**, 529-539, doi:10.1007/BF0023370 (1990).

37 Itano, K., Kawai, S., Miyazaki, N., Tatsukawa, R. & Fujiyama, T. Mercury and selenium levels in striped dolphins caught off the Pacific Coast of Japan. *Agric. Biol. Chem.* **48**, 1109-1116, doi:10.1271/bbb1961.48.1109 (1984).

38 Kehrig, H. A., Hauser-Davis, R. A., Seixas, T. G., Pinheiro, A. B. & Di Beneditto, A. P. M. Mercury species, selenium, metallothioneins and glutathione in two dolphins from the southeastern Brazilian coast: mercury detoxification and physiological differences in diving capacity. *Environ. Pollut.* **213**, 785-792, doi:10.1016/j.envpol.2016.03.041 (2016).

39 Wagemann, R., Stewart, R. E. A., Béland, P. & Desjardins, C. Heavy metals in tissues of beluga whales from various locations in the Canadian Arctic and the St. Lawrence River. *Can. Bull. Fish. Aquat. Sci./Bull. Can. Sci. Halieut. Aquat.* **224**, 191-206 (1990).

40 Wagemann, R., Trebacz, E., Boila, G. & Lockhart, W. L. Methylmercury and total mercury in tissues of arctic marine mammals. *Sci. Total Environ.* **218**, 19-31, doi:10.1016/s0048-9697(98)00192-2 (1998).

41 Paludan-Muller, P., Agger, C. T., Dietz, R. & Kinze, C. C. Mercury, cadmium, zinc, copper and selenium in harbour porpoise (*Phocoena phocoena*) from West Greenland. *Polar Biol.* **13**, 311-320, doi:10.1007/BF0023835 (1993).

42 Bezerra, M. F., Lacerda, L. D., Lima, E. & Melo, M. T. D. Monitoring mercury in green sea turtles using keratinized carapace fragments (scutes). *Mar. Pollut. Bull.* **77**, 424-427, doi:10.1016/j.marpolbul.2013.09.020 (2013).

43 Faust, D. R. *et al.* Inorganic elements in green sea turtles (Chelonia mydas): Relationships among external and internal tissues. *Environ. Toxicol. Chem.* **33**, 2020-2027, doi:10.1002/etc.2650 (2014).

44 Peterson, S. A., Van Sickle, J., Herlihy, A. T. & Hughes, R. M. Mercury concentration in fish from streams and rivers throughout the western united states. *Environ. Sci. Technol.* **41**, 58-65, doi:10.1021/es061070u (2007).

45 Gingerich, P. D. Body Weight and Relative Brain Size (Encephalization) in Eocene Archaeoceti (Cetacea). *J. Mamm. Evol.* **23**, 17-31, doi:10.1007/s10914-015-9304-y (2016).

46 Crile, G. & Quiring, D. P. A record of the body weight and certain organ and gland weights of 3690 animals. *The Ohio Journal of Science* **40**, 219-259 (1940).

47 Kastelein, R. & Vaughan, N. Food consumption, body measurements and weight changes of a female killer whale (*Orcinus orca*). *Aquat. Mamm.* **15**, 18-21 (1989).

48 Lockyer, C. Body weights of some species of large whales. *ICES J. Mar. Sci.* **36**, 259-273, doi:10.1093/icesjms/36.3.259 (1976).

49 Gallo-Reynoso, J. P. & Figueroa-Carranza, A. L. Size and weight of Guadalupe fur seals. *Mar. Mamm. Sci.* **12**, 318-321, doi:10.1111/j.1748-7692.1996.tb00584.x (1996).

50 Godsell, J. The relative influence of age and weight on the reproductive behaviour of male grey seals Halichoerus grypus. *J. Zool.* **224**, 537-551, doi:10.1111/j.1469-7998.1991.tb03784.x (1991).

51 Hamilton, J. E. Weight, etc., of elephant seal. *Nature* **163**, 536-536, doi:10.1038/163536b0 (1949).

52 FAO. Food and Agriculture Organization of the United Nation (FAO). FAOSTAT statistics database. Available online at http://fao.org/faostat. (2017).
